# The Effect of Aromatic Diimide Side Groups on the π-Conjugated Polymer Properties

**DOI:** 10.3390/polym10050487

**Published:** 2018-05-01

**Authors:** Anna Drewniak, Mateusz D. Tomczyk, Lukasz Hanusek, Anna Mielanczyk, Krzysztof Walczak, Pawel Nitschke, Barbara Hajduk, Przemyslaw Ledwon

**Affiliations:** 1 Faculty of Chemistry, Silesian University of Technology, Strzody 9, 44-100 Gliwice, Poland; anna.drewniak@polsl.pl (A.D.); Mateusz.D.Tomczyk@polsl.pl (M.D.T.); lhanusek95@gmail.com (L.H.); anna.mielanczyk@polsl.pl (A.M.); krzysztof.walczak@polsl.pl (K.W.); 2 Centre of Polymer and Carbon Materials, Polish Academy of Sciences, Curie-Sklodowskiej 34, 41-819 Zabrze, Poland; PNitschke@cmpw-pan.edu.pl (P.N.); bhajduk@cmpw-pan.edu.pl (B.H.)

**Keywords:** π-conjugated polymer, donor-acceptor, perylenediimide, PDI, naphtalenediimide, NDI

## Abstract

The presented study describes the method for the synthesis and characterization of a new class of conjugated copolymers containing a perylenediimide (PDI) and naphthalene diimide (NDI) side groups. The main conjugated backbone is a donor-acceptor polymer poly[3,6-carbazole-*alt*-5,5-(4′,7′-di-2-thienyl-2′,1′,3′-benzothiadiazole)] containing thiophene and carbazole as donor units and benzothiadiazole as an acceptor unit. The presented compounds were synthesized in a multistep synthesis. The polymerization was carried out by Suzuki or Stille coupling reaction. Redox properties of the studied polymers were tested in different conditions. Electrochemical investigation revealed independent reduction of the main polymer chain and diimide side groups. UV-Vis spectroscopy revealed the overlap of two absorption spectra. The difference between the electron affinity of the polymer main chain and that of the diimides estimated electrochemically is approximately 0.3 eV.

## 1. Introduction

One of the best perspective research fields of π-conjugated polymers is organic photovoltaics (OPV) [1]. The operation of solar cells is possible through photogeneration of charge carriers in a semiconductor material, and their consecutive separation and transport to the respective electrodes through the active layer [2,3,4]. Early organic solar cells utilized primarily bilayer architectures, whereas currently bulk heterojunction (BHJ) devices have become preeminent among these devices. 

The BHJ requires donor and acceptor types of organic semiconductors to be combined. Small molecules, conjugated oligomers, or polymers can be used. The typical photoactive blend layer in BHJ OPV cells consist of conjugated polymer donor and fullerene derivative as molecular acceptor [5]. However, recent years bring the intense development of a new class of BHJ solar cells with nonfullerene acceptor molecules [5]. Diimide derivatives constitute one of the most promising groups among nonfullerene-based acceptors [5]. Perylenediimide (PDI) and naphthalenediimide (NDI) derivatives are among the most intensively studied groups of diimiides [6,7,8,9]. PDI, NDI, and their derivatives are of particular interest due to their unique properties such as high chemical, thermal, and photochemical stability, large optical absorption, significant electron transport properties, and wide modification possibilities [10]. PDI derivatives are one of the most stable organic compounds with the π-conjugated system [11]. This results in their widespread use as industrial pigments for automotive paints and textile coloration. Currently, PDI and NDI derivatives are widely tested materials as n-type semiconductors [12,13]. According to literature data, there are no efficient solar cells with simple, low molecular PDI or NDI units. However, modification of PDI units by incorporation of additional aromatic side groups [14], combining of few PDI units [15,16,17], or the incorporation of side atoms stiffening the structure [18,19] can be an effective way to significantly improve the performance of organic solar cells up to 8.4%. There are examples of polymers with PDI in the main polymer chain [10,20]. However, the main drawback of such compounds is a complex purification of intermediate brominated PDI derivatives. 

Organic photovoltaics-based BHJ requires solving many problems. One of the main identified issues is the stability of organic materials. There are some reports which focus on this, maybe the most important issue [21]. Another important issue is the constancy of layer morphology. During operation, solar cells are exposed to elevated temperatures. Over time, this often leads to phase separation of acceptor and donor components leading to the considerable decrease in the device performance. This is one of the reasons which limits the use of organic solar cells on a larger scale. A short exciton diffusion length is one of the main issues occurring in BHJ. After light absorption formed exciton must migrate to the donor–acceptor interface and split into free charge carriers. This phenomenon requires fitting of HOMO and LUMO energy levels of the donor and acceptor molecules and the short distance of these materials. The performance of an acceptor-based small molecule is largely limited by the decrease in electron mobility and recombination losses [22].

A single-component BHJ structure is one of the strategies which can lead to resolving the important issue of phase separation and charge separation of organic solar cells with simultaneous maintenance of the short distance between donor and acceptor moieties. Those studies focused on PDI as acceptor. These polymers consist of typical donor-type backbone and PDI side chain. Examples of donor-acceptor polymer architecture including polymers with PDI in the side chain were reported [23,24,25,26,27,28,29]. The results of BHJ OPV based on these polymers show the efficiency typically much less than 1%. However, recent research shows that such structures have great potential leading to devices with good efficiency exceeding 4% [30,31]. Moreover, according to our knowledge, there are no reported polymers with NDI side groups employed as the electron acceptor. For these reasons, it is extremely important to deepen the knowledge about such materials.

**Scheme 1:**
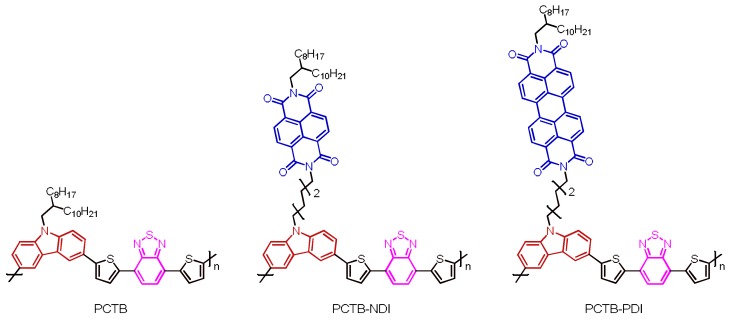
Structures of studied polymers.

Electrochemical methods can be valuable to identify both stability and electronic properties as well as the charge formation in new material. Electrochemical methods do not provide direct results, but they can be useful methods to estimate these extremely important properties. Our polymers consist of the donor–acceptor type of polymers with two acceptors (Scheme 1), benzothiadiazole in the main chain and PDI or NDI derivatives in the side chain. Such a structure leads to a single-component BHJ. The research includes a comparison of the synthesis and properties of three polymers designed to evaluate the effects of two acceptors in the polymer chain: first acceptor benzothiadiazole, which is involved in conjugation in the polymer backbone and is common for all compounds, and second PDI or NDI acceptor in the nonconjugated side chain. They were compared in terms of the absorption of light, electrochemical properties, energy levels, and photovoltaic effect. 

## 2. Materials and Methods

### 2.1. Synthesis

**Scheme 2:**
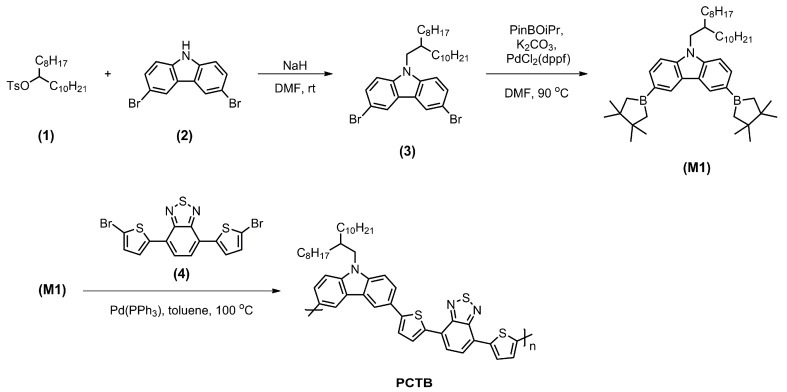
Synthesis path for polymer PCTB.

**Scheme 3:**
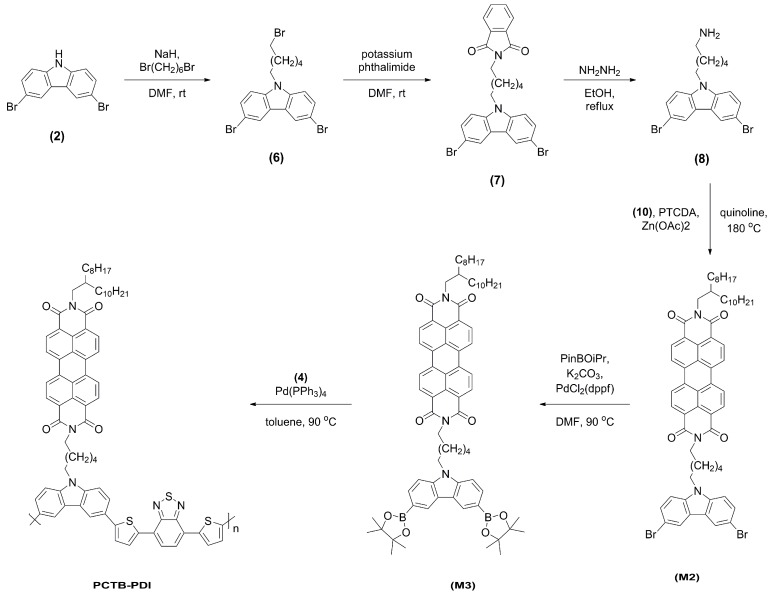
Synthesis path for polymer PCTB-PDI.

**Scheme 4:**
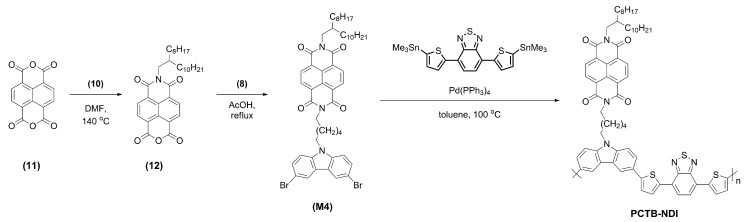
Synthesis path for polymer PCTB-NDI.

Polymers PCTB, PCTB-PDI, and PCTB-NDI were synthesized as shown in Scheme 2, Scheme 3 and Scheme 4. Starting compounds and other reagents were provided by Sigma-Aldricht (Saint Louis, MO, USA), TCI (Nihonbashi-honcho, Japan), Acros Organics (Thermo Fisher Scientific, Geel, Belgium), or Fluorochem (Hadfield, UK) unless otherwise stated. Syntheses of compounds 6, 7, 8 were made according to modification of procedures from ref. [32]. Polymerizations of PCTB and PCTB-PDI were made according to a standard Suzuki coupling procedure adapted from ref. [27]. Polymerization of PCTB-NDI was made according to a standard Stille coupling procedure adapted from ref. [33]. 

#### 2.1.1. 3,6-Dibromo-9-(2-octyldodecanyl)-9H-carbazole (3)

3,6-Dibromo-9H-carbazole (1.0 g, 3.1 mmol) and anhydrous DMF (5 mL) were charged into a dry flask and stirred. When 3,6-dibromo-9H-carbazole was dissolved, 60% NaH in mineral oil (0.18 g, 4.5 mmol) was added. After 30 min, 2-octyldodecyl 4-methylbenzenesulfonate was added. The mixture was stirred overnight at room temperature. The solution was poured into water and extracted with DCM (3 × 30 mL). The organic layer was dried over anhydrous MgSO_4_. The solution was filtered and evaporated. The crude product was purified by column chromatography (silica gel) eluting with DCM-hexane (1:1 *v*/*v*) resulting as colourless oil (1.7 g; Yield 78.0%). ^1^H NMR (300 MHz, CDCl_3_) δ 8.14 (d, *J* = 1.8 Hz, 2H), 7.54 (dd, *J* = 8.6, 1.8 Hz, 2H), 7.25 (d, *J* = 8.6 Hz, 2H), 4.09 (d, *J* = 7.6 Hz, 2H), 2.04 (m, 1H), 1.34–1.15 (m, 32H), 0.91–0.80 (m, 6H). ^13^C NMR (75 MHz, chloroform-*d*) δ 139.88, 129.09, 123.53, 123.30, 112.06, 110.78, 48.15, 37.98, 32.06, 32.02, 31.98, 29.99, 29.86, 29.73, 29.71, 29.63, 29.58, 29.46, 29.36, 26.69, 22.84, 22.79, 14.27, 14.25. HRMS (*m*/*z*): 605.2056 [M], 607.2046 [M + 2].

#### 2.1.2. Monomer M1

Compound **3** (460 mg, 0.76 mmol), bis(pinacolato)diboron (771 mg, 3.04 mmol), potassium acetate (447 mg, 4.56 mmol), PDCl_2_(dppf) (37 mg, 0.05 mmol), and anhydrous DMF (10 mL) were charged into a dry Schlenk flask and degassed. The solution was stirred at 80 °C for 21 h. The solution was poured into water and extracted with DCM (3 × 30 mL). The organic layer was washed with water and brine, and then dried over anhydrous MgSO_4_. The solution was filtered and evaporated. The crude product was purified by column chromatography (silica gel) eluting with DCM-hexane (1:1) to give a product as a colourless oil (284 mg; Yield: 61%). ^1^H NMR (300 MHz, CDCl_3_) δ 8.66 (s, 2H), 7.89 (dd, *J* = 8.2, 1.2 Hz, 2H), 7.36 (d, *J* = 8.2 Hz, 2H), 4.16 (d, *J* = 7.4 Hz, 2H), 2.10 (br, 1H), 1.61–1.19 (m, 32H), 0.92–0.82 (m, 6H). ^13^C NMR (75 MHz, chloroform-*d*) δ 143.14, 131.93, 127.98, 122.80, 108.41, 83.50, 47.83, 37.80, 31.91, 31.85, 29.90, 29.88, 29.59, 29.55, 29.48, 29.32, 29.22, 26.56, 26.54, 24.96, 22.69, 22.65, 14.12.

#### 2.1.3. 3,6-Dibromo-9-(6-bromohexyl)-9H-carbazole (6)

3,6-Dibromo-9H-carbazole (2.0 g, 6.2 mmol) and anhydrous DMF (25 mL) were charged into a dry flask and stirred. When 3,6-dibromo-9H-carbazole was dissolved, 60% NaH in mineral oil (0.246 g, 6.2 mmol) was added. After 30 min, 1,6-dibromohexane (3.0 g, 12.4 mmol) was added. The mixture was stirred overnight at room temperature. The solution was poured into water (75 mL) and extracted with 5 × 50 mL of EtOAc. The organic layer was washed with water (6 × 100 mL) and dried over anhydrous Na_2_SO_4_. The solution was filtered and evaporated. The crude product was purified by recrystallization from hexane to give a product as a white solid (1.18 g; Yield: 39%). ^1^H NMR (300 MHz, CDCl_3_) δ 8.13 (s, 2H), 7.55 (d, *J* = 6.0 Hz, 2H), 7.23 (d, *J* = 6.0 Hz, 2H), 4.24 (t, *J* = 6.6 Hz, 2H), 3.36 (t, *J* = 6.6 Hz, 2H), 1.80 (quin, *J* = 6.6 Hz, 2H), 1.51–1.29 (m, 6H). ^13^C NMR (75 MHz, chloroform-*d*) δ 139.15, 128.95, 123.35, 123.18, 111.92, 110.21, 43.01, 33.50, 32.35, 28.59, 27.65, 26.26. HRMS (*m*/*z*): 486.8955 [M], 488.8940 [M + 2].

#### 2.1.4. 2-(6-(3,6-Dibromo-9H-carbazol-9-yl)hexyl)isoindoline-1,3-dione (7)

Compound **6** (1.13 g, 2.32 mmol) was dissolved in DMF (7 mL). The solution was stirred and cooled in an ice bath under Ar atmosphere. Potassium phthalimide (813 mg, 4.3 mmol) was added in one portion. After 15 min, the ice bath was removed and the solution was stirred overnight at room temperature. The solution was poured into water (100 mL) and filtered. The precipitate was dried under vacuum, dissolved in 100 mL of DCM, and absorbed on silica gel. The product was purified by column chromatography eluting with DCM-hexane (1:2) to give a product as a white solid (1.02 g, 79.0%). ^1^H NMR (300 MHz, CDCl_3_) δ 8.11 (s, 2H), 7.83 (dd, *J* = 6.0, 3.0 Hz, 2H), 7.71 (dd, *J* = 6.0, 3.0 Hz, 2H), 7.53 (d, *J* = 6.0 Hz, 2H), 7.24 (d, *J* = 6.0 Hz, 2H), 4.22 (t, *J* = 6.6 Hz, 2H), 3.64 (t, *J* = 6.6 Hz, 2H), 1.84–1.57 (m, 4H), 1.39–1.32 (m, 4H). ^13^C NMR (75 MHz, chloroform-*d*) δ 168.37, 139.14, 133.84, 132.26, 128.92, 123.14, 123.10, 123.06, 111.87, 110.23, 43.13, 37.62, 28.56, 28.26, 26.61, 26.39. HRMS (*m*/*z*): 563.5530 [M], 565.5671 [M + 2].

#### 2.1.5. 6-(3,6-Dibromo-9H-carbazol-9-yl)hexyl-1-amine (8)

Compound **7** (980 mg, 1.8 mmol), hydrazine (1.3 mL), and ethanol (36 mL) were charged into the flask with a reflux condenser. The solution refluxed for 4 h. After cooling to room temperature, the reaction mixture was filtered. The crude product was purified by recrystallization from ethanol to give a product as a white solid (640 mg, Yield: 85.0%). ^1^H NMR (300 MHz, DMSO-*d6*) δ 8.09 (d, *J* = 1.8, 2H), 7.88 (d, *J* = 6.0 Hz, 2H), 7.57 (d, *J* = 6.0 Hz, 2H), 4.39 (t, *J* = 6.6 Hz, 2H), 3.02 (t, *J* = 6.6 Hz, 2H), 1.76–1.34 (m, 2H), 1.44–1.37 (m, 2H), 1.33–1.20 (m, 4H). ^13^C NMR (75 MHz, DMSO-*d6*) δ 131.59, 125.58, 123.87, 123.33, 112.01, 110.45, 50.79, 42.93, 31.16, 28.82, 27.12, 18. HRMS (*m*/*z*): 425.0052 [M + H]^+^, 427.0030 [M + 2 + H]^+^.

#### 2.1.6. Monomer M2

2-octyldodecan-1-amine (**10**; 196 mg, 0.71 mmol), Compound **8** (280 mg, 0.71 mmol), Zn(OAc)_2_ (152 mg, 0.83 mmol), perylene-3,4,9,10-tetracarboxylic acid anhydride (**PTCDA**; 277 mg, 0.71 mmol and quinoline (7 mL) were charged into the dry flask. The solution was stirred at 180 °C for 4 h. The solution was cooled, poured into MeOH (14.5 mL), and 2 M HCl solution (20 mL) was added. The solution was stirred overnight. The obtained suspension was filtered and the resulting precipitate was washed with water and MeOH. The precipitate was dried under vacuum, dissolved in 60 mL of chlorobenzene, and absorbed on silica gel. The product was purified by column chromatography eluting with DCM to give a product as a red solid (240 mg, 32%). ^1^H NMR (600 MHz, CDCl_3_) δ 8.59 (d, *J* = 7.8 Hz, 2H), 8.53 (d, *J* = 7.8 Hz, 2H), 8.47 (d, *J* = 7.8 Hz, 2H), 8.44 (d, *J* = 7.8 Hz, 2H), 7.99 (d, *J* = 1.8 Hz, 2H), 7.48 (dd, *J* = 8.4, 1.8 Hz 2H), 7.22 (d, *J* = 8.4 Hz, 2H), 4.21 (t, *J* = 7.2 Hz, 2H), 4.18–4.11 (m, 4H), 2.03–1.97 (m, 1H), 1.85 (t, *J* = 7.2 Hz, 2H), 1.73 (t, *J* = 7.2 Hz, 2H), 1.51–1.15 (m, 36 H), 0.89–0.78 (m, 6H). ^13^C NMR (150 MHz, chloroform-*d*) δ 163.61, 163.25, 148.50, 139.24, 139.09, 134.48, 134.26, 131.31, 131.21, 129.25, 129.16, 129.01, 126.21, 123.38, 123.34, 123.15, 123.05, 122.88, 111.96, 110.34, 77.23, 77.01, 76.80, 44.78, 43.30, 40.37, 36.68, 31.93, 31.91, 31.79, 30.08, 29.67, 29.65, 29.60, 29.35, 29.32, 28.67, 27.76, 26.93, 26.74, 26.55, 22.68, 14.11. HRMS (*m*/*z*): 699.5645 [M], 700.5664 [M + H]^+^.

#### 2.1.7. Monomer M3

Compound **M2** (130 mg, 0.121 mmol), bis(pinacolato)diboron (122 g, 0.482 mmol), potassium acetate (7.1 mg, 0.724 mmol), and anhydrous DMF (2 mL) were charged into a 15 mL dry flask and purged with Ar. After 30 min, PdCl_2_(dppf) (6 mg, 0.007 mmol) was added. The solution was stirred at 80 °C for 1 h and then at 90 °C for 24 h. The solution was cooled to room temperature and poured into water (15 mL). Product was extracted by DCM (10 mL and 3 × 4 mL). The separated organic layer was washed with water (13 mL) and brine (10 mL), then dried over MgSO_4_. The solution was filtered and solvent was removed under vacuum. The crude product was purified by column chromatography eluting with DCM-MeOH (199:1) to give a product as a red solid (65 mg, Yield: 46%). ^1^H NMR (300 MHz, CDCl_3_) δ 8.62 (s, 2H), 8.60 (s, 2H), 8.51 (s, 2H), 8.48 (s, 2H), 7.87 (dd, *J* = 8.2, 1.2 Hz, 2H), 7.36 (d, *J* = 8.2 Hz, 2H), 7.20 (s, 2H), 4.30 (t, *J* = 6.6 Hz, 2H), 4.16 (t, *J* = 6.6 Hz, 2H), 4.12 (d, *J* = 7.2 Hz, 2H), 2.01–1.82 (m, 1H), 1.57–1.37 (m, 4H), 1.34–1.20 (m, 60 H), 0.87–0.80 (m, 6H). ^13^C NMR (75 MHz, chloroform-*d*) δ 164.02, 163.92, 143.28, 134.74, 134.52, 132.08, 128.12, 126.78, 124.42, 124.32, 123.52, 123.32, 123.19, 123.08, 122.95, 108.55, 83.68, 45.02, 43.54, 36.71, 31.90, 31.74, 31.04, 30.13, 30.03, 29.63, 29.46, 29.34, 28.78, 27.72, 26.80, 26.69, 26.50, 25.05, 24.99, 22.65, 14.08.

#### 2.1.8. N-(2-Octyldodecyl)naphthalene-1,8-dicarboxyanhydride-4,5-dicarboxyimide (12)

1,4,5,8-naphthalenetetracarboxylic dianhydride (901 mg, 3.36 mmol) and DMF (6 mL) were charged into the dry flask. The solution was purged by Ar and stirred at 120 °C for 30 min. Then the solution of 2-octyldodecan-1-amine (800 mg, 2.69 mmol) in DMF (14 mL) was added dropwise over 4 h at 120 °C. The solution was stirred at 140 °C for 16 h. Then the solution was chilled at −20 °C for 24 h. The formed suspension was filtered. The crude product was purified by column chromatography eluting with DCM to give a product as a brownish solid (188 mg, 13%). ^1^H NMR (300 MHz, CDCl_3_) δ 8.88–8.84 (m, 4H), 4.18 (d, *J* = 7.2, 2H), 2.07–1.94 (m, 1H), 1.41–1.20 (m, 28H), 0.95–0.84 (m, 12H). ^13^C NMR (75 MHz, chloroform-*d*) δ 163.62, 163.07, 133.30, 131.41, 129.76, 128.04, 125.17, 45.35, 36.74, 32.04, 32.01, 31.79 31.77, 30.12, 29.79, 29.75, 29.72, 29.68, 29.47, 29.41, 26.53, 22.81, 22.79, 14.24.

#### 2.1.9. Monomer M4

Compound **12** (168 mg, 0.307 mmol), Compound **8** (0.130 g; 0.307 mmol), and glacial acetic acid (20 mL) were mixed together and refluxed for 24 h. The solution was heated under refluxing condition for 24 h, then cooled to the room temperature and poured into water. The suspension was centrifuged. The crude product was purified by column chromatography eluting with DCM and twice recrystallized from EtOAc-MeOH (1:1) to give a product as a yellow solid (79 mg, Yield: 27%). ^1^H NMR (600 MHz, chloroform-*d*) δ 8.72 (d, *J* = 7.2 Hz, 2H), 8.70 (d, *J* = 7.2 Hz, 2H), 8.06 (s, 2H), 7.50 (d, *J* = 6.0 Hz, 2H), 7.25 (d, *J* = 6.0 Hz, 2H), 4.24 (t, *J* = 6.6 Hz, 2H), 4.17–4.10 (m, 4H), 2.02–1.93 (m, 1H), 1.90–1.80 (m, 2H), 1.75–1.62 (m, 2H), 1.48–1.15 (m, 36H), 0.90–0.80 (m, 6H). ^13^C NMR (150 MHz, chloroform-*d*) δ 163.12, 162.78, 139.22, 130.95, 130.91, 129.00, 126.64, 126.39, 123.38, 123.20, 111.96, 110.32, 77.20, 76.99, 76.78, 44.98, 43.21, 40.59, 36.59, 31.89, 31.85, 31.64, 29.98, 29.60, 29.60, 29.57, 29.52, 29.31, 29.26, 28.63, 27.78, 26.84, 26.66, 26.41, 22.66, 22.63, 14.10.

#### 2.1.10. Polymer PCTB

Monomer **M1** (140 mg, 0.20 mmol), 4,7-bis(2-bromo-5-thienyl)-2,1,3-benzothiadiazole (**4**) (91.7 mg, 0.20 mmol), Aliquat 336 (20 mg), toluene (2.8 mL), and 20% aqueous K_2_CO_3_ (1.7 mL) were charged into a Schlenk flask purged with Ar for 30 min. Tetrakis(triphenylphosphine)palladium (0) (Pd(PPh_3_)_4_, 2.3 mg, 0.002 mmol) was added to the solution. The solution was frozen, degassed, and refilled with Ar; this procedure was repeated twice. The solution was heated to 90 °C and stirred. After 3 days, iodobenzene (0.1 mL) in additional degassed 5 mL of toluene was added. After 12 h phenyl boronic acid was added. After 2 h the mixture was cooled to the room temperature and poured into 50 mL of MeOH. The impurities were removed in Soxhlet apparatus using MeOH and then hexane. Further, the polymer was extracted using CHCl_3_. The solution of polymer in CHCl_3_ was reduced under the vacuum and the polymer was again precipitated in MeOH. The residue was filtered using a nylon filter. The dark purple polymer was obtained (150 mg, Yield: 94%). ^1^H NMR (600 MHz, CDCl_3_) δ 8.44–6.05 (m, 12H), 4.24–3.72 (m, 2H), 2.34–2.29 (m, 1H), 1.67–1.05 (m, 32H), 0.95–0.75 (m, 6H). GPC: M_n_= 2208 g/mol, M_w_= 2567 g/mol.

#### 2.1.11. Polymer PCTB-PDI

Compound **M3** (50 mg, 0.043 mmol), 4,7-bis(2-bromo-5-thienyl)-2,1,3-benzothiadiazole (20 mg, 0.043 mmol), Aliquat 336 (40 mg), toluene (3.5 mL), and 20% aqueous K_2_CO_3_ (0.5 mL) were charged into a 15 mL Schlenk flask which was purged with Ar for 30 min. Pd(PPh_3_)_4_ (1 mg, 0.001 mmol) was added to the solution. The solution was frozen, degassed, and refilled with Ar; this procedure was repeated twice. The solution was heated to 90 °C and stirred. After 3 days iodobenzene (0.1 mL) in additionally degassed 5 mL of toluene was added. After 12 h phenyl boronic acid was added. After 2 h the mixture was cooled to the room temperature and poured into 50 mL of MeOH. The impurities were removed in Soxhlet apparatus using MeOH and hexane. Further, the polymer was extracted using CHCl_3_. The solution of polymer in CHCl_3_ was reduced under the vacuum and the polymer was again precipitated in MeOH. The residue was filtered using a nylon filter. The dark purple product was obtained (44 mg, Yield: 84%). ^1^H NMR (600 MHz, CDCl_3_) δ 8.78–8.45 (m, 8H), 8.11–8.05 (m, 2H), 7.56–7.25 (m, 4H), 7.30–7.25 (m, 6H), 4.27–4.06 (m, 6H), 1.90–1.12 (m, 41H), 0.91–0.78 (m, 6H). GPC: M_n_ = 2907 g/mol, M_w_ = 4360 g/mol.

#### 2.1.12. Polymer PCTB-NDI

Compound **M4** (80 mg, 0.058 mmol) and toluene (3.5 mL) were charged into a Schlenk flask, which was purged with Ar for 30 min. 4,7-Bis(5-trimethylstannyl-2-thienyl)-2,1,3-benzothiadiazole (36 mg, 0.058 mmol) and Pd(PPh_3_)_4_ (1 mg, 0.002 mmol) were added into the mixture. The mixture was frozen, degassed, and refilled with Ar; this procedure was repeated twice. The mixture was heated to 100 °C and stirred. After 3 days iodobenzene (0.1 mL) was added. After 2 h the mixture was cooled to the room temperature and poured into 60 mL of MeOH. The impurities were removed in Soxhlet apparatus using MeOH, hexane, and acetone. The polymer was then extracted using CHCl_3_ and then chlorobenzene. Solvents were evaporated. Polymer was dissolved in CHCl_3_ and precipitated in MeOH. The obtained product was dried to the constant mass. The dark purple product was obtained (58 mg, Yield: 92.0%). ^1^H NMR (600 MHz, CDCl_3_) δ 8.89–8.34 (m, 4H), 8.17–8.03 (m, 2H), 7.81–7.08 (m, 10H), 4.77–4.61 (m, 2H), 4.40–3.51 (m, 4H), 2.11–0.99 (m, 41H), 0.91–0.70 (m, 6H). M_n_ = 2163 g/mol, M_w_ = 2473 g/mol.

### 2.2. Characterization Techniques

NMR spectra were recorded at 600 MHz for ^1^H NMR and 150 MHz for ^13^C NMR on a Varian Mercury Plus 600 MHz spectrometer (Palo Alto, CA, USA) or at 300 MHz for ^1^H and 75 MHz for ^13^C NMR on a Varian XL-300 spectrometer. 

The UV-Vis spectroscopy of the polymer samples was carried out in 0.1 mg/mL CHCl_3_ solution and in the form of a polymer layer deposited by spin coating on the glass plate. The solvent used in the analysis of the polymer solution, CHCl3 chloroform, was pure for analysis. The measurements were carried out on a UV-Vis HP 8453 spectrophotometer in Hellma absorption cell with a 2 mm light path. 

Electrochemical measurements included cyclic voltammetry of polymers in the form of solid films. The measurement was performed in a classic three-electrode system using the Autolab Metrohm PGSTAT100N potentiostat (Utrecht, The Netherlands). The working electrode was the ITO electrode, the auxiliary electrode was a platinum spiral, while the pseudo-reference electrode was a silver electrode. The potential was estimated using ferrocene as a standard. A solution of 0.1 M tetrabutylammonium hexafluorophosphate in acetonitrile was used as the supporting electrolyte. The electrolyte was purged with argon to remove oxygen from the system. 

Molecular weights and dispersity indices (Ð) were determined by size-exclusion chromatograph (SEC, Agilent Technologies, Santa Clara, CA, USA) equipped with an 1100 Agilent 1260 Infinity isocratic pump, autosampler, degasser, thermostatic box for columns, and differential refractometer MDS RI Detector. Addon Rev. B.01.02 data analysis software (Agilent Technologies, Santa Clara, CA, USA) was used for data collecting and processing. The SEC calculated molecular weights were based on calibration applying linear polystyrene standards (Mp = 580–327,000 g/mol). Pre-column guard 5 µm (50 × 7.5 mm) and PLGel 5 mm MIXED-D (300 × 7.5 mm) column were used for separation. 

### 2.3. Device Preparation and Characterization

Devices with one component bulk heterojunction were prepared on ITO Glass Substrates (Ossila, 6 pixels, Sheffield, UK) with an active area of 4.5 mm^2^. Devices were fabricated by spin coating of PEDOT:PSS film and then polymer film on ITO. Solutions of polymers were obtained by dissolving desired polymers in chlorobenzene. IV-curves of photovoltaic devices were measured by PV Test Solutions Solar Simulator (Wroclaw, Poland) under AM1.5 solar illumination and Keithley 2400. Thickness of thin layers was controlled with the use of variable-angle spectroscopic ellipsometry. The SENTECH SE850E spectroscopic ellipsometer (Berlin, Germany) is working in the spectral range 240–2500 nm, under the Spectra Ray 3 Software (Berlin, Germany).

## 3. Results and Discussion

### 3.1. Synthesis 

The structure of polymers is based on the same main chain, which is composed of thiophene, carbazole, and benzothiadiazole units. The thiophene and carbazole units act as electron donors, and the benzothiadiazole units are electron acceptors. Polymers have side chains attached to the carbazole nitrogen atoms. The bulky side chains of 2-octanedodecyl group were used to enable solubility of polymers. The PCTB polymer has a 2-octanedodecyl group as a side chain, while the other two polymers have additional electron-accepting units in the side chains PCTB-PDI and PCTB-NDI connected by the hexyl linker. Monomers were synthetized by modification of procedure from ref. [32]. 

Polymers were obtained with good yields. Obtained SEC results indicate receiving rather low molecular weights compounds/oligomers with *M*_n_ below 3000 g/mol. However, additional SEC analysis performed for comparative purposes indicates that *M*_n_ values estimated using polystyrene standards are apparently significantly understated. SEC analysis of an analogue oligomer with an absolute molecular weight 2136 g/mol was performed (please see Supplementary Information Figure S3d). The resulting number average molecular weight was 1156 g/mol which is almost twice lower than the absolute molecular weight of this oligomer. The difference between the relative and absolute molecular weights probably stems from the distinct polymeric structures having different hydrodynamic volumes of studied samples and polystyrene standards. Thus, *M*_n_ values are provided only to compare the obtained polymers with each other. It can also be assumed that the real *M*_n_ is almost twice as large as that determined with the use of polystyrene standards.

### 3.2. Physicochemical Properties

**Figure 1:**
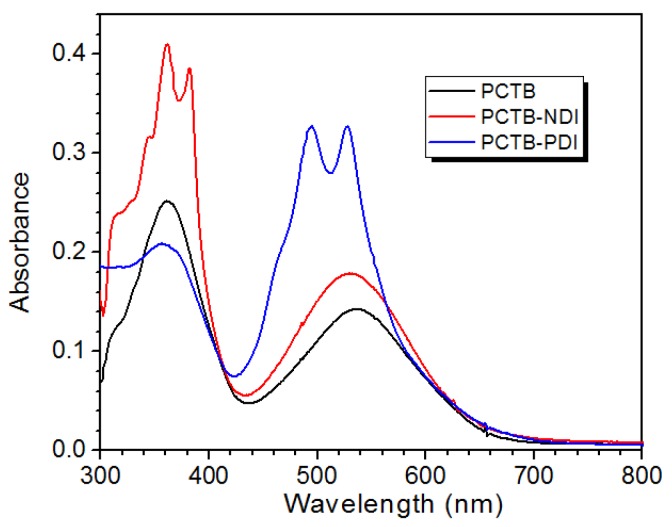
UV-Vis spectra of polymer solutions in CHCl_3_.

The UV−Vis absorption spectra of polymer solutions with a concentration of 0.1 mg/mL in CHCl_3_ are shown in Figure 1. CHCl_3_ was chosen due to good solubility of polymers in this solvent. All polymers present broad and strong absorption in the visible and near ultraviolet range. Results are summarized in Table 1.

In the UV−Vis absorption spectrum of PCTB the broad transition with the maximum at 538 nm with the maximum mass attenuation coefficient 14.2 L∙g^−1^∙cm^−1^ is observed. This peak corresponds to the intramolecular charge transfer which is typically observed in the donor-acceptor type of polymers [34,35]. The absorption in the near ultraviolet range is even stronger with maximum mass attenuation coefficient 25.4 L∙g^−1^∙cm^−1^ at 361 nm.

The UV−Vis absorption spectra of PCTB-NDI reveal some important differences. The peak corresponding to the intramolecular charge transfer of PCTB-NDI is slightly shifted in comparison to PCTB. The maximum absorption is at wavelength 530 nm with mass attenuation coefficient 15.1 L∙g^−1^∙cm^−1^. It can be related to the different geometry of main polymer backbone as branched alkyl groups of PCTB have been replaced by hexyl linkers in PCTB-NDI. The analysis of near ultraviolet range reveals the complex character of light absorption with a double peak having its maxima at 362 and 382 nm. The mass attenuation coefficient is 34.4 and 32.5 L∙g^−1^∙cm^−1^, respectively. These transitions are derived from the light absorption of the NDI units and the polymer main chain.

In the case of PCTB-NDI spectrum the overlap of absorption transitions of PDI unit and polymer backbone is already observed in the visible range. A strong absorption results in several distinct peaks with two main absorption maxima at 494 and 527 nm with 31.4 and 31.3 L∙g^−1^∙cm^−1^, respectively. In the near ultraviolet range maximum at 357 nm with 20.0 L∙g^−1^∙cm^−1^ is observed. The lower absorption than PCTB is related to the lower concentration of polymer main chain compared to PCTB and low absorption of PDI units in this range.

### 3.3. Electrochemical Properties

**Figure 2:**
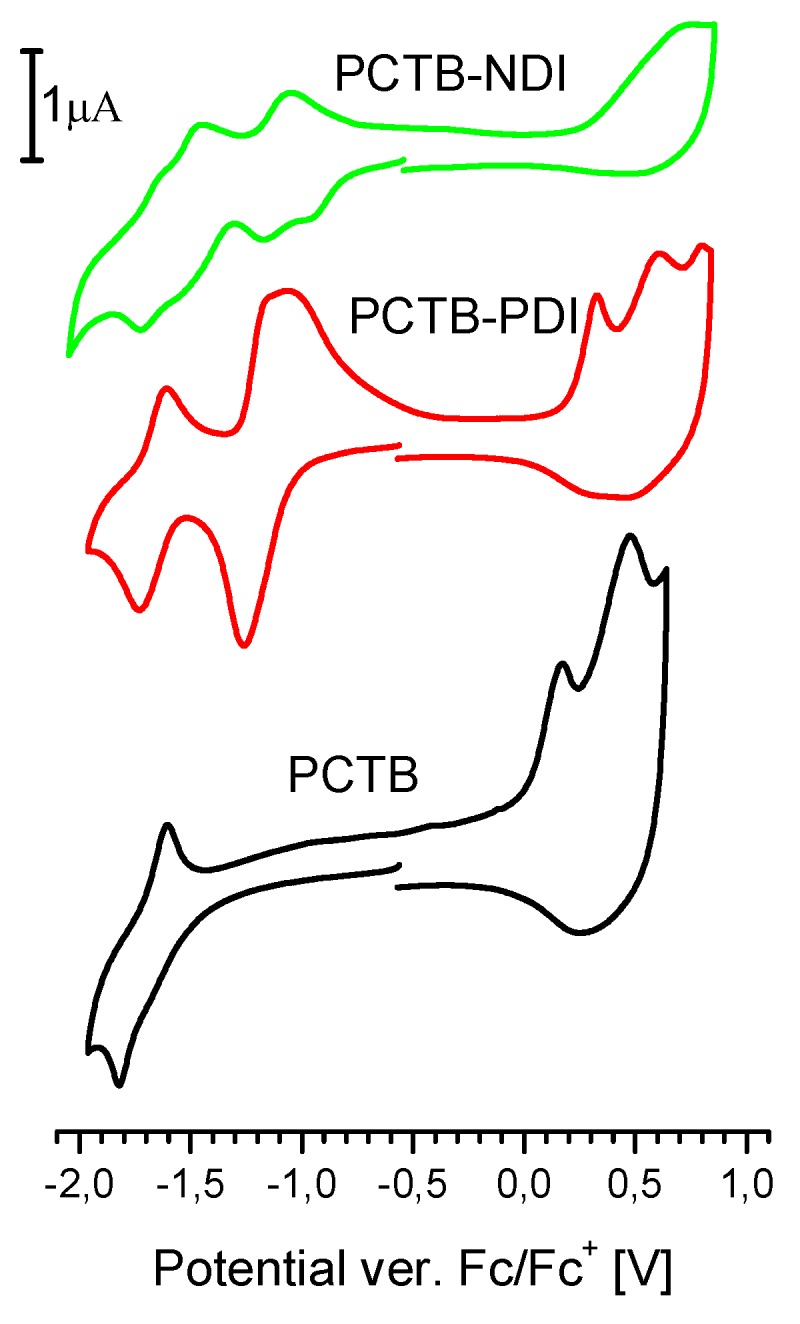
Cyclic voltammetry of polymer films spin coated on ITO electrode with ACN/Bu_4_NBF_4_ as supporting electrolyte.

Electrochemical properties of all polymers were estimated by cyclic voltammetry of solid films immersed to an ACN/Bu_4_NBF_4_ as electrolyte. Cyclic voltammetry curves shown in Figure 2 exhibit reversible oxidation in the cathodic range and reduction in the anodic range. 

The reduction of polymers was analyzed in detail. Cyclic voltammetry of PCTB reveals the reduction onset potential at −1.41 V and reduction redox pair at potential −1.82/−1.60 V. The onset potential of the first reduction peak of PCTB-PDI is −1.04 V. The reduction peak at −1.27 V is related to the two-electron process of PDI reduction. The two-electron nature of this peak is particularly observed at counter peak. In this case the peak separation is clearly observed. In the lower potential range another reduction redox pair at −1.72/−1.60 V is observed. This potential is similar to the position of the redox pair of PCTB. This indicates the connection of this process with reduction of the main polymer chain. 

The cyclic voltammetry curve of PCTB-NDI reveals at least three redox pairs in the cathodic range. The reduction onset potential of PCTB-NDI is located at −0.85 V and the first reduction redox pair is located at −1.16/−1.04 V. The second and third reduction redox pairs are located at −1.52/−1.45 and −1.71/−1.62 V, respectively. The potentials of the first and second peaks fit the NDI reduction, the third with main chain reduction.

The reduction of PCTB-PDI and PCTB-NDI is more complex than PCTB. In both cases, additional reduction peaks are observed as expected. It indicates the independent reduction of PDI or NDI units and the main polymer backbone. Aromatic diimides are well-known compounds with a low energy level of LUMO orbital. Bezothiadiazole is a strong electron-acceptor unit whose incorporation to the polymer backbone leads to the donor-acceptor type of material. The combination of these two different electron-acceptor units leads to the bulk heterojunction with separated redox moieties. This, in turn, can be beneficial for application as one component, polymer-based heterojunction.

The oxidation of studied polymers shows only slight differences. The oxidation of PCTB begins at about 0 V, while for PCTB-PDI and PCTB-NDI it is slightly shifted to higher potentials. Charge trapping is observed at voltammetry of PCTB and PCTB-PDI observed as the peak at the onset of oxidation. This effect is not observed for PCTB-NDI. Charge trapping is a common phenomenon which occurs in thin solid films of π-conjugated films during electrochemical n- and p-type of dedoping [36,37]. The occurrence of this phenomenon can be caused by poor electric conductivity of conjugated polymers in their neutral state or structural defects. Its intensity increases with increasing layer thickness and also depends on such elements as memory effect during multiple electrochemical doping end dedoping.

### 3.4. Electron Paramegnetic Resonance Spectroelectrochemistry of PCTB-PDI

**Figure 3:**
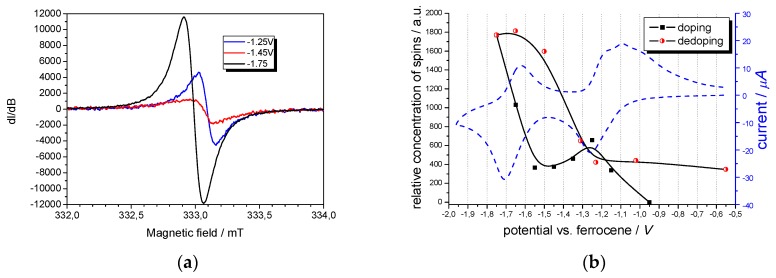
Electron Paramagnetic Resonance spectroelectrochemistry: (**a**) first derivative of selected EPR signals in restricted range; (**b**) relative concentration of spins at different potential.

The reduction process of PCTB-PDI was studied by Electron Paramagnetic Resonance (EPR). Measurement was made in situ during electrochemical reduction of polymer film in the potentiostatic conditions. Selected EPR signals are shown in Figure 3a. At the potential related to the first oxidation peak, the EPR signal appeared. The relative concentration of spins was estimated and related to the cyclic voltammetry curve of the polymer as shown in Figure 3b.

The appearance of the EPR signal at the beginning of polymer reduction is related to the formation of spin-bearing radical anions located at PDI unit. The further change of potential in the negative direction leads to a decrease of EPR signal as a result of the formation of spinless dianions. 

In the potential related to the reduction of the main polymer chain, a new additional strong signal is observed. The signal is shifted in comparison to the first signal. It indicates different surroundings of these two types of radical. The second one is related to the formation of radical anions localized on the benzothiadiazole moiety [36]. It clearly shows that reduction processes of the main polymer chain and PDI units are separated.

### 3.5. Photovoltaic Properties

**Figure 4:**
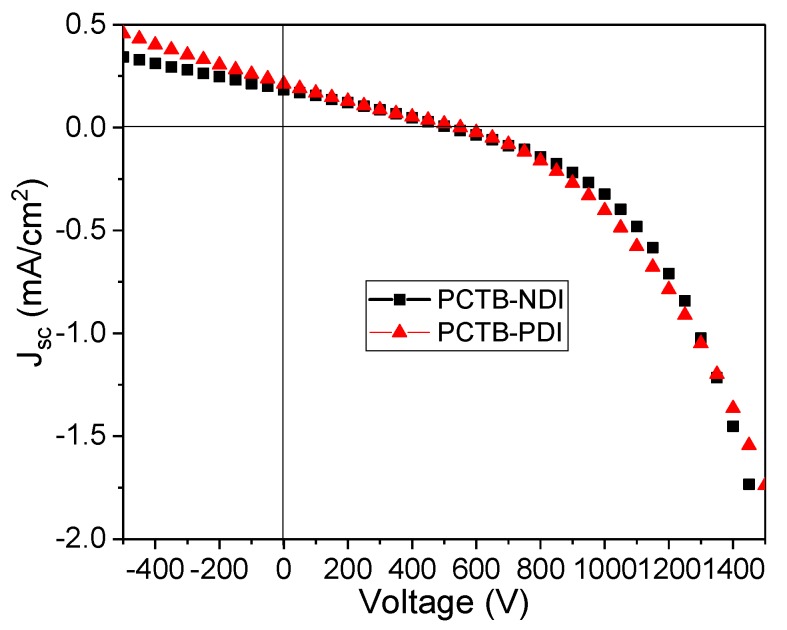
IV-curves of photovoltaic devices with PCTB-NDI or PCTB-PDI as one-component BHJ.

Photovoltaic properties of studied compounds were tested with the use of one component bulk heterojunction with structure ITO/PEDOT:PSS/PCTB-NDI/Al and ITO/PEDOT:PSS/PCTB-PDI/Al. In this heterojunction, the main PCTB polymer chain acts as an electron donor and diimides are electron acceptors. IV-curves of photovoltaic devices measured under AM1.5 solar illumination are presented in Figure 4.

Open circuit voltage V_oc_ of the device with PCTB-NDI active layer is 0.521 V and the device with PCTB-PDI is 0.54 V. Such values indicate good matching of HOMO and LUMO energy levels of donor main chain and acceptor diimide side groups.

The power conversion efficiency of both devices is 0.03%. IV-curves indicate the issue with charge carrier transport in BHJ. Probably it is the consequence of arrangements of PDI units related to hindered π-stacking ability of side PDI or NDI moieties due to the presence of long branched alkyl groups which is a common phenomenon in such types of acceptors [38]. Obtained results indicate the need to modify the structure of polymers to improve charge carriers transport to electrodes.

## 4. Conclusions

In summary electrochemical and spectroelectrochemical investigation reveals the independent reduction of the main polymer chain and diimide side groups. The charge carriers localized on π-conjugated main polymer chains and aromatic diimide side groups are separated. Independent absorption of diimide side groups and main polymer chains was observed as the overlap of absorption bands. The PDI units have already been used in analogous structures in combination with other main polymers chains. Our study shows that the use of NDI is also possible. Moreover, NDI units show differences in comparison to PDI which in some cases can be beneficial. The use of NDI units leads to the material with shifted Electron Affinity up to 4.2 eV, separated two-step reduction process of diimide moiety, and absorption in the ultraviolet part of spectra. Electron affinity of PCTB-PDI value is 4.1 eV, the two-electron reduction of diimide moieties is almost simultaneous, and the maximum absorption occurs in the visible part of spectra. Energy levels of HOMO and LUMO orbitals of PCTB-NDI and PCTB-PDI are well fitted for BHJ solar cells. However, the weak performance of solar cells indicates the need for further modification of the obtained structures in order to improve the transport of the charge carriers. 

## Figures and Tables

**Table 1:** The summary of photophysical, electrochemical, and thermal properties.

Compound	λ ^1^ [nm]	E_ox_^2^ [V]	E_red_^3^ [V]	IP ^4^ [eV]	EA ^5^ [eV]	T_g_^6^ [°C]
PCTB	352; 535	0	−1.41	5.1	3.8	140
PCTB-NDI	361; 382; 521	0.26	−0.85	5.4	4.2	
PCTB-PDI	359; 494; 527	0.18	−1.04	5.3	4.1	155

^1^ Absorption maxima; ^2^ Oxidation onset potential; ^3^ Reduction onset potential; ^4^ Ionization potential estimated from equation IP = |e^−^|(5.1 + Eox); ^5^ Electron affinity estimated from equation EA = |e^−^|(5.1 + Ered); ^6^ Glass transition temperature.

## Supplementary Materials

The following are available online at http://www.mdpi.com/2073-4360/10/5/487/s1.
