# Supplementary material for: The Effect of Aromatic Diimide Side Groups on the π-Conjugated Polymer Properties
Source: Polymers (Basel). 2018 May 1;10(5):487. doi: 10.3390/polym10050487 (PMC6415361; doi:10.3390/polym10050487)
Supplement: Supplementary file 1 [file polymers-10-00487-s001.pdf]

Supplementary information

The effect of aromatic diimide side groups on the π-conjugated polymer properties

Anna Drewniak[1], Mateusz D. Tomczyk[1], Lukasz Hanusek[1], Anna Mielanczyk[1], Krzysztof Walczak[1], Pawel Nitschke[2], Barbara Hajduk[2] and Przemyslaw Ledwon[1]

[1]Silesian University of Technology, Faculty of Chemistry, 44-100 Gliwice, Strzody 9, Poland; krzysztof.walczak@polsl.pl

[2]Centre of Polymer and Carbon Materials, Polish Academy of Sciences, 41-819 Zabrze, Curie-Skłodowskiej 34, Poland; bhajduk@cmpw-pan.edu.pl

[*]Correspondence: przemyslaw.ledwon@polsl.pl; Tel.: +48-32-237-1305

Contents

1. NMR data P. 1-5
2. DSC data P. 6,7
3. SEC data P. 8-11
4. AFM data P. 12

M1-^{1}H NMR

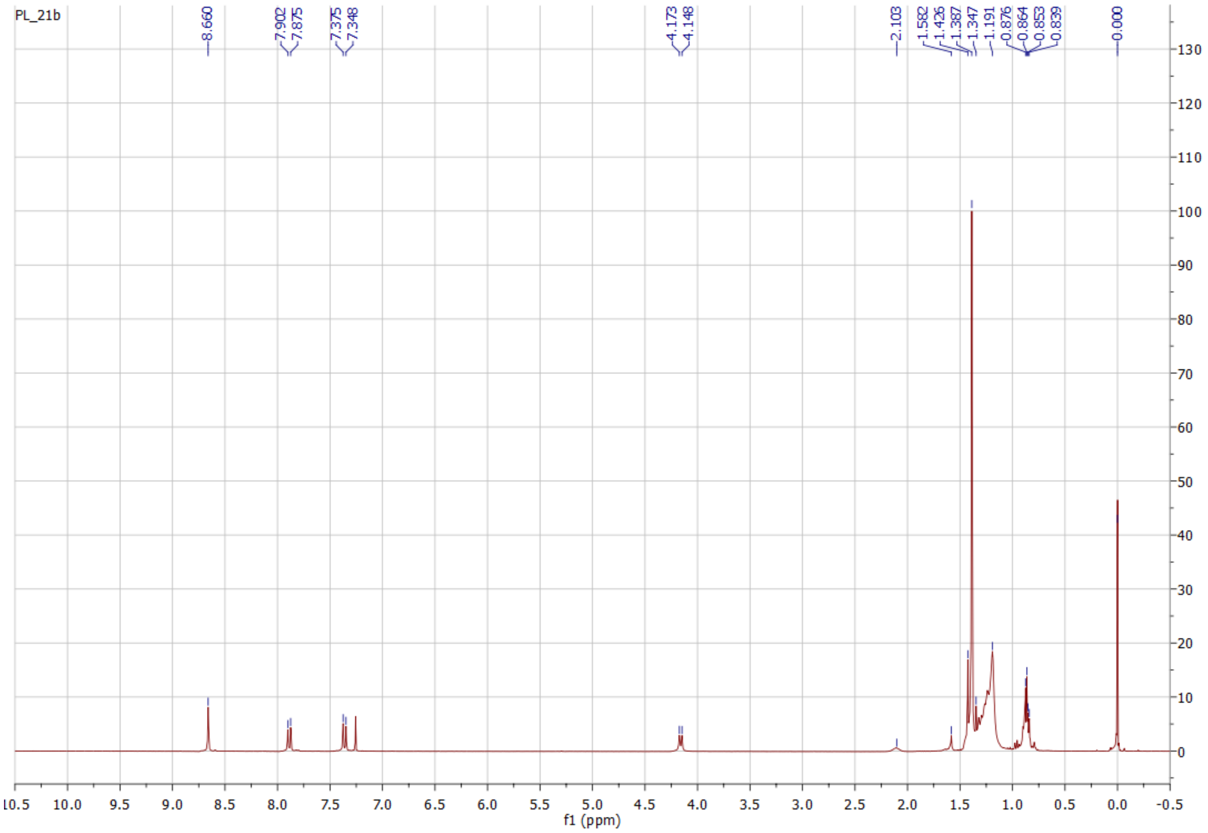

M1-^{13}C NMR

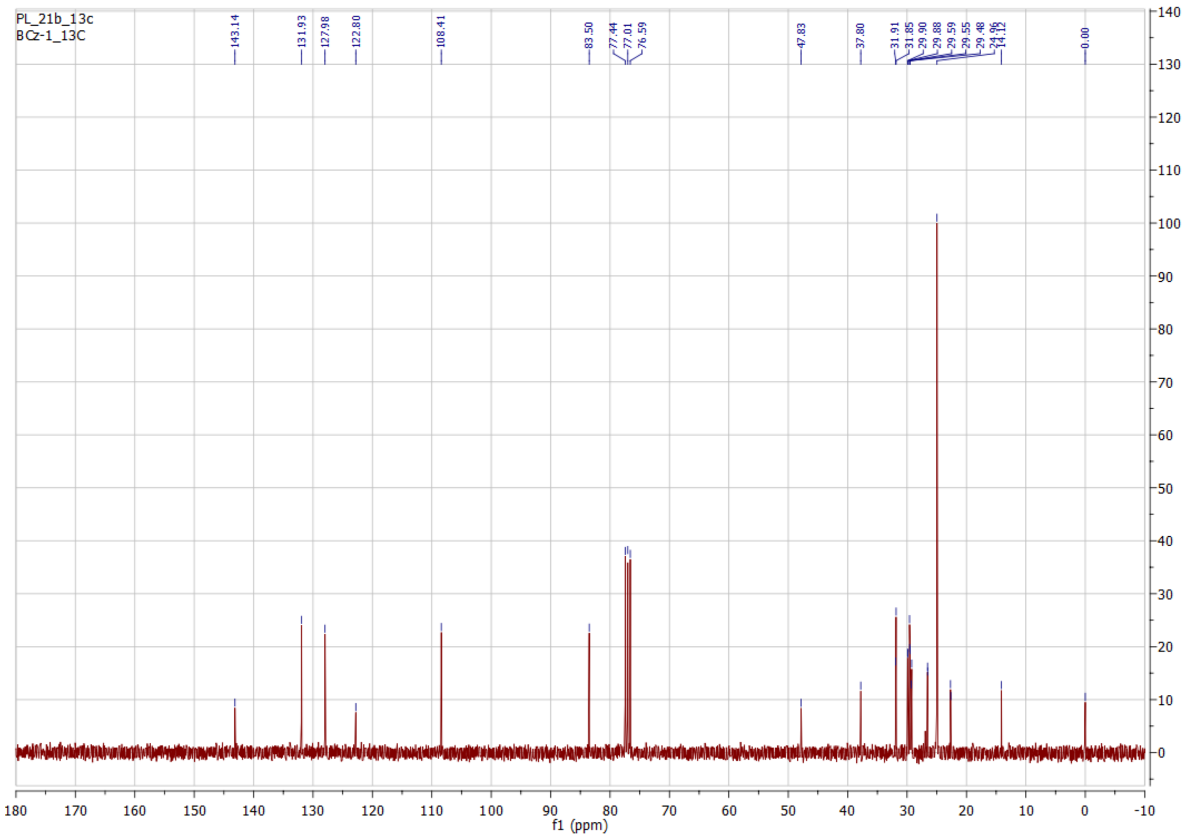
PL_21b_13c
BCz-1_13C
143.14
131.93
127.98
122.80
108.41
83.50
77.44
77.01
76.59
47.83
37.80
31.91
31.85
29.90
29.88
29.59
29.55
29.48
14.12
0.00
f1 (ppm)

PCTB-^{1}H NMR

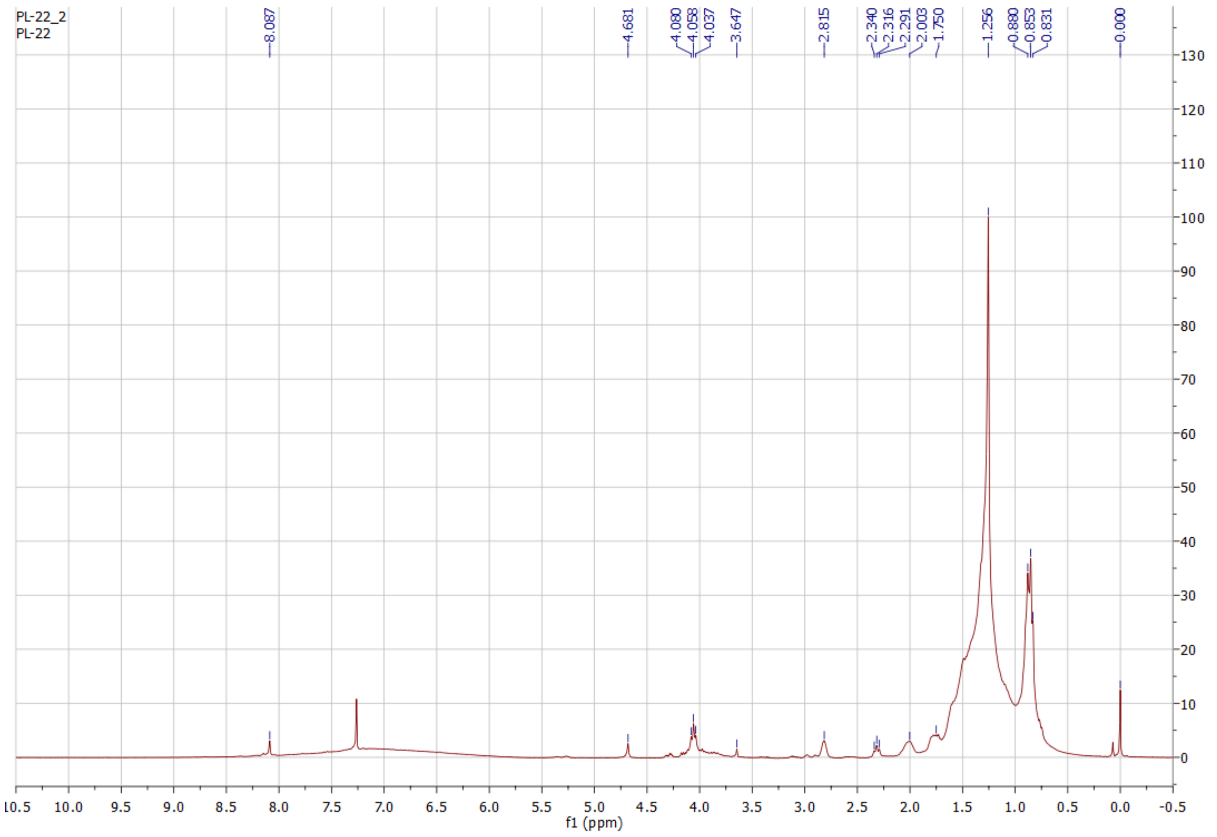
PL-22_2
PL-22
8.087
4.681
4.080
4.058
4.037
3.647
2.815
2.340
2.316
2.291
2.003
1.750
1.256
0.880
0.853
0.831
0.000
f1 (ppm)

M2-1H

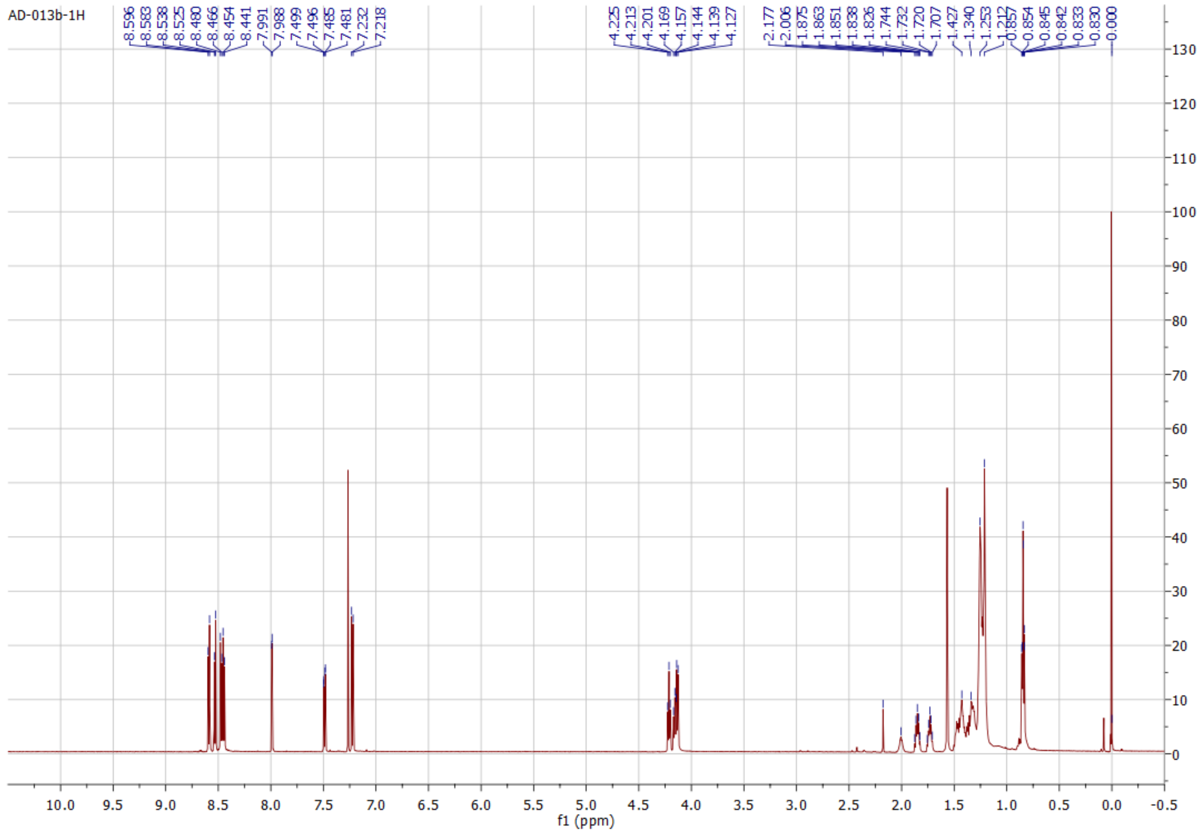

M2-^{13}C NMR

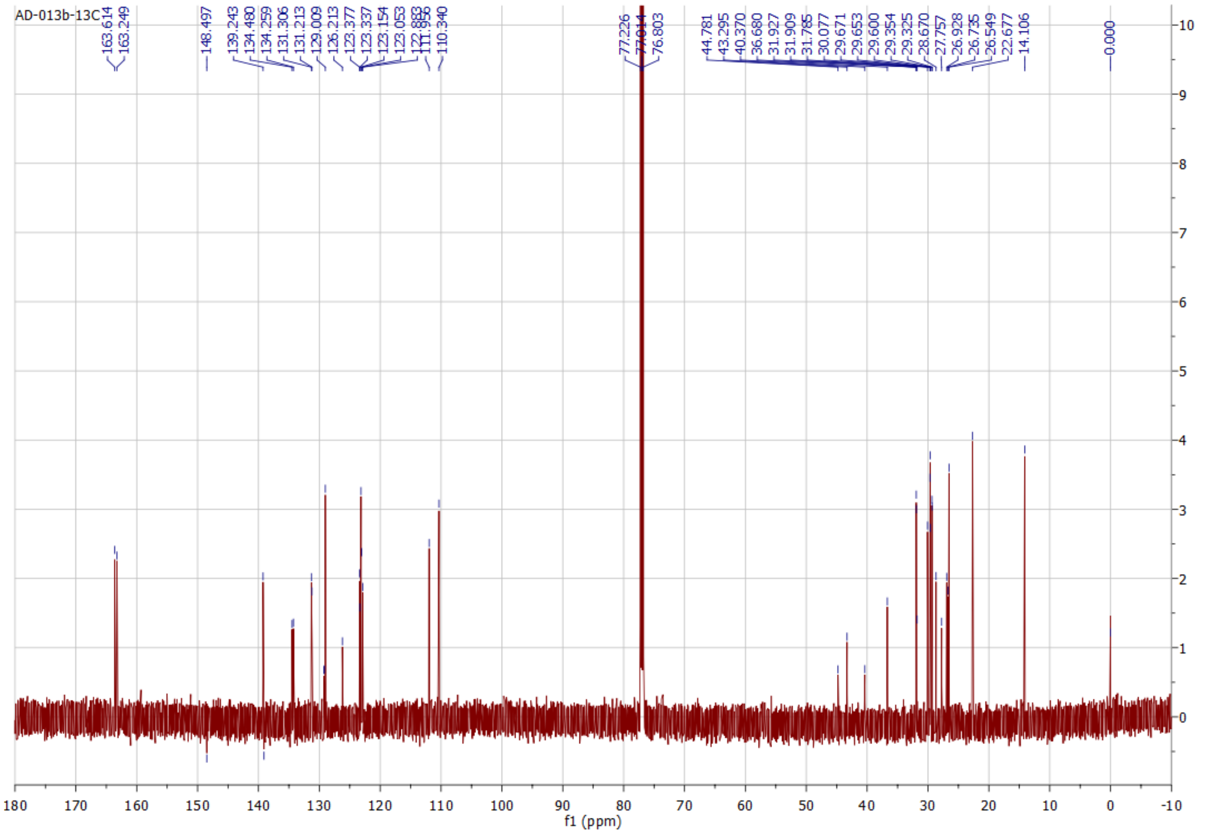

PCTB-PDI-^{1}H NMR

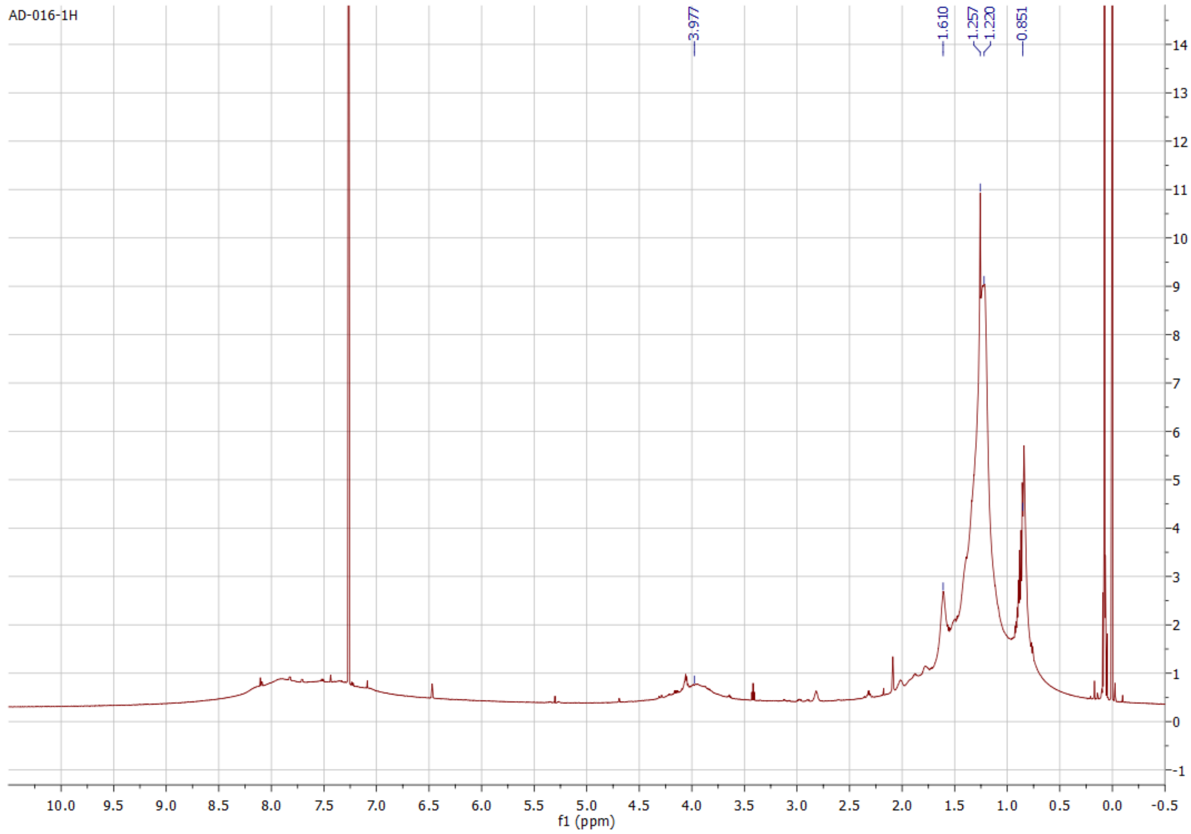
AD-016-1H
3.977
1.610
1.257
1.220
0.851
f1 (ppm)

M4-^{1}H NMR

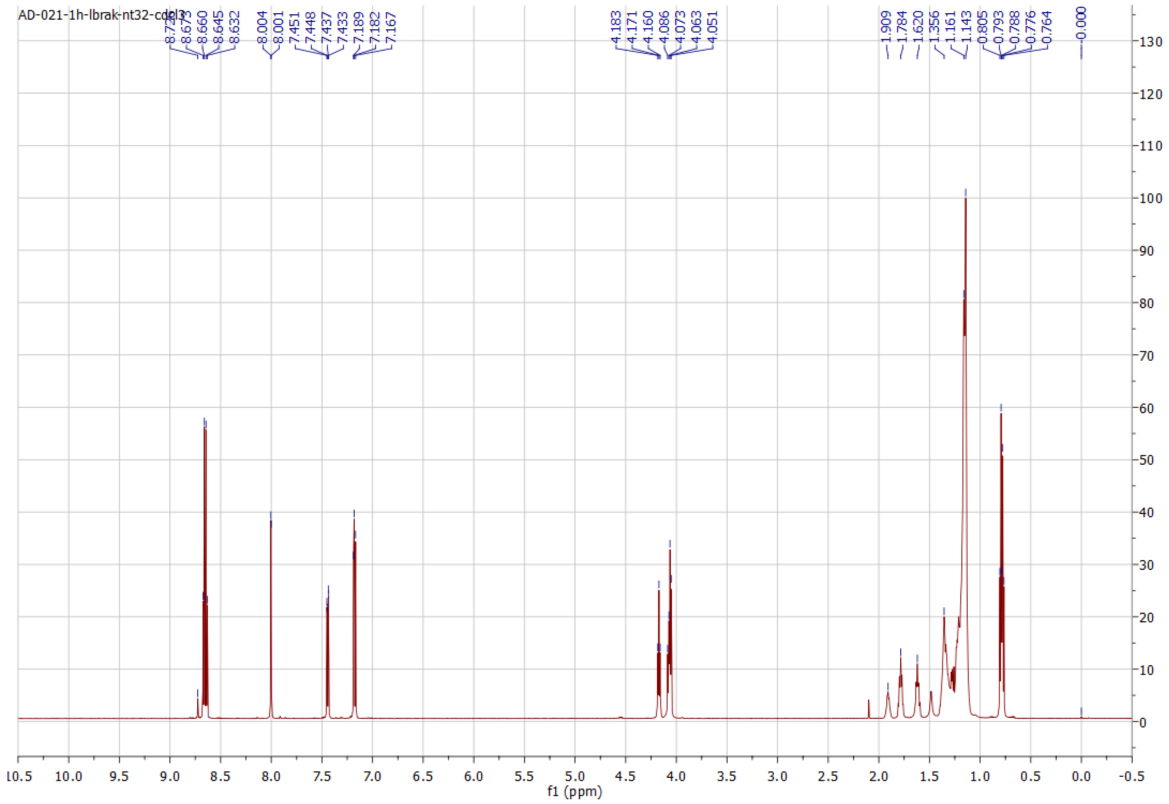
AD-021-1h-lbrak-nt32-cdcl3
8.673
8.660
8.645
8.632
8.004
8.001
7.451
7.448
7.437
7.433
7.189
7.182
7.167
4.183
4.171
4.160
4.086
4.073
4.063
4.051
1.909
1.784
1.620
1.356
1.161
1.143
0.805
0.793
0.788
0.776
0.764
0.000
f1 (ppm)

M4-^{13}C NMR

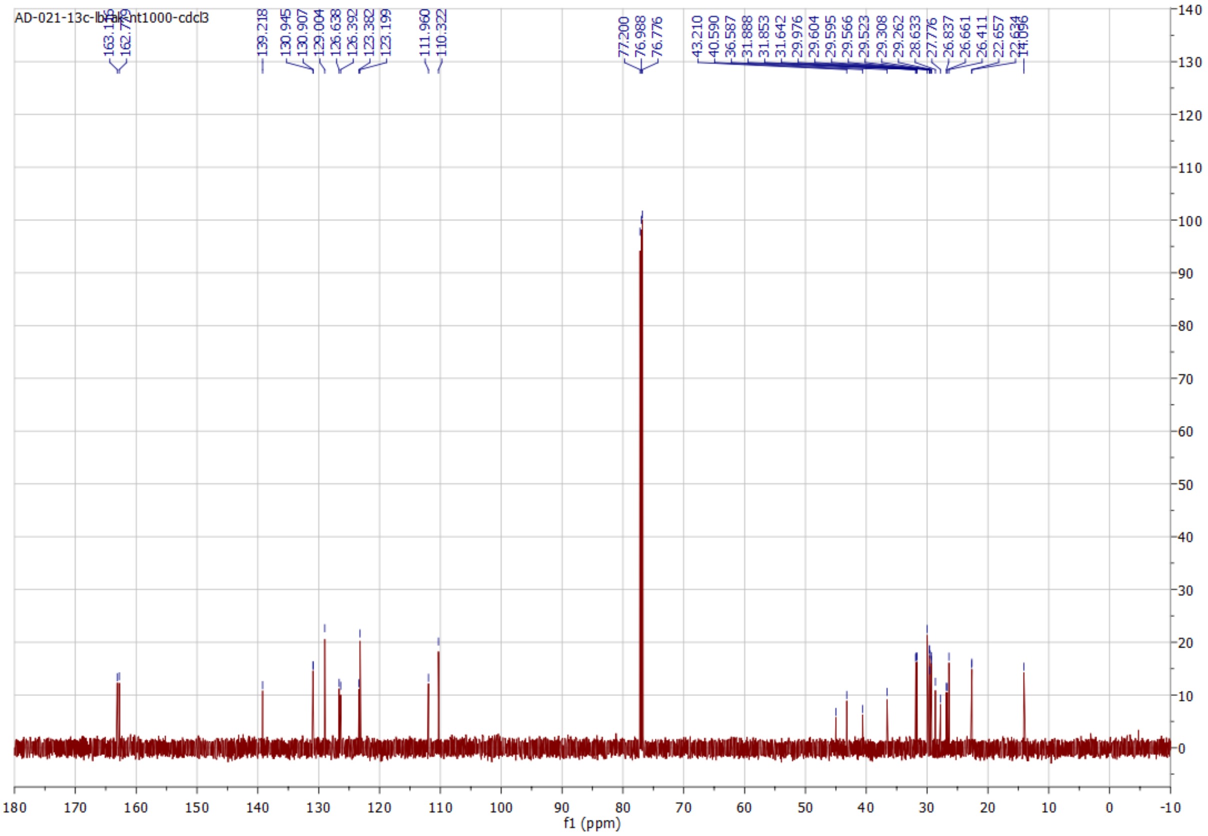

PCTB-NDI-^{1}H NMR

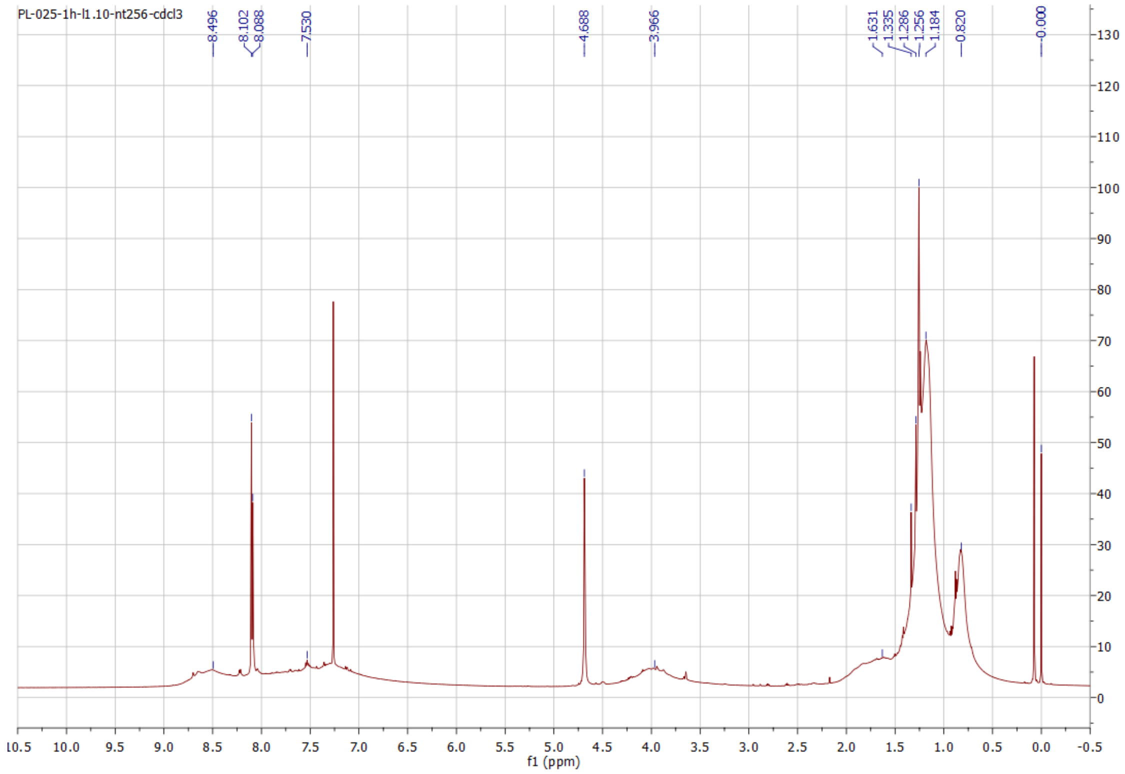

Fig. S1 ^{1}H NMR and ^{13}C NMR spectra

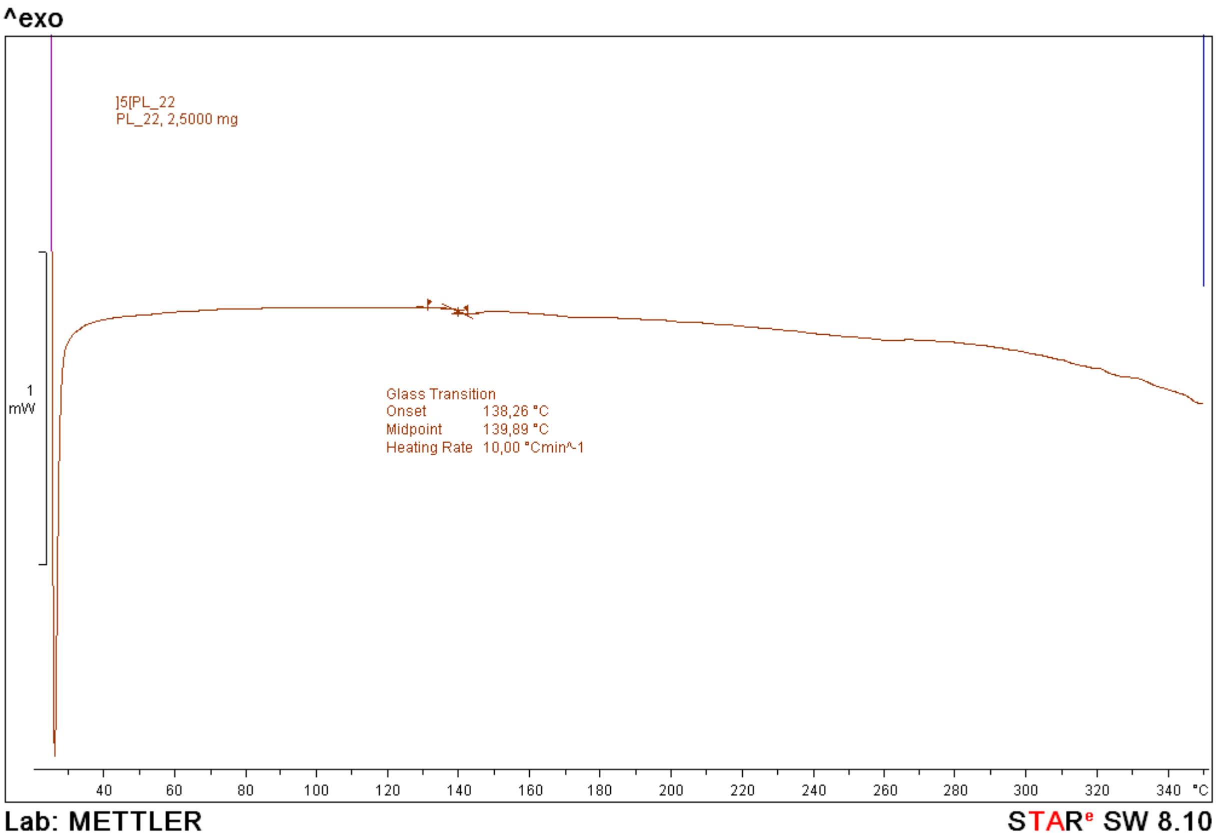

Fig. S2a DSC of PCTB

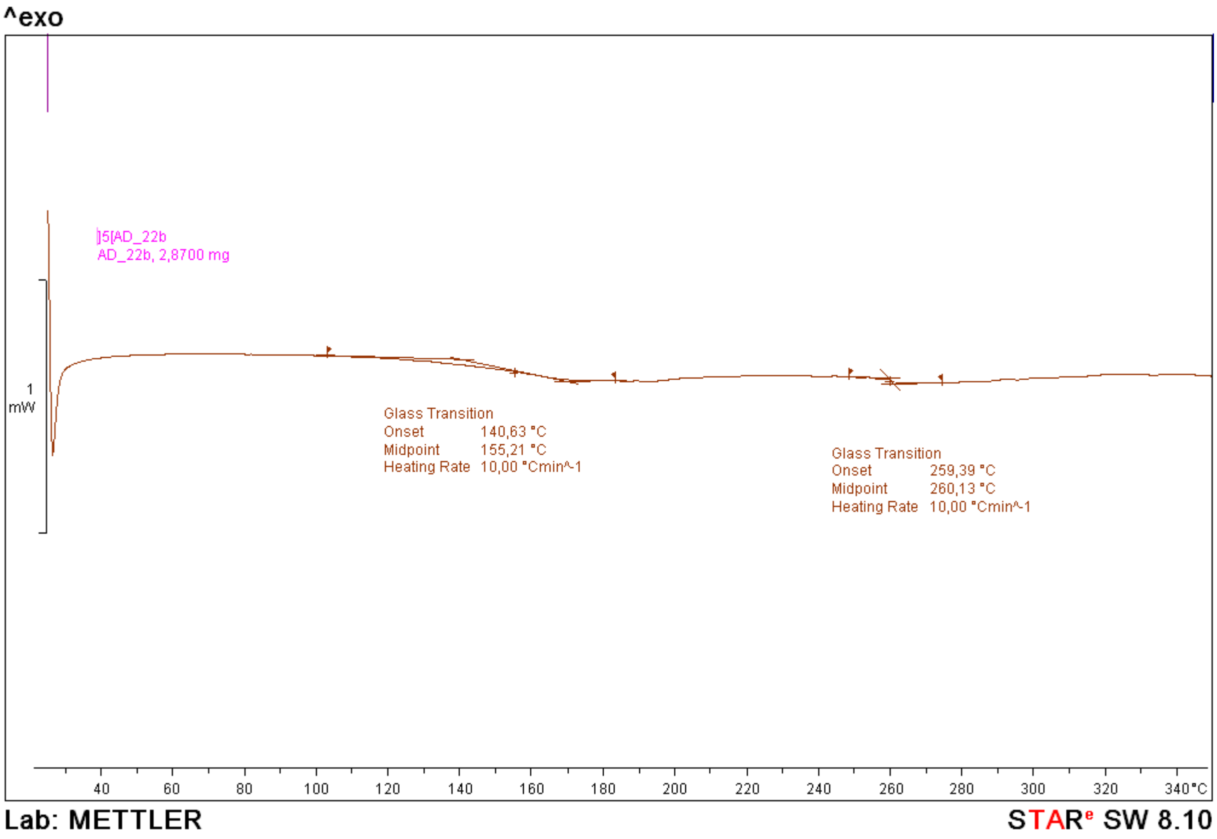

Fig. S2b DSC of PCTB-PDI

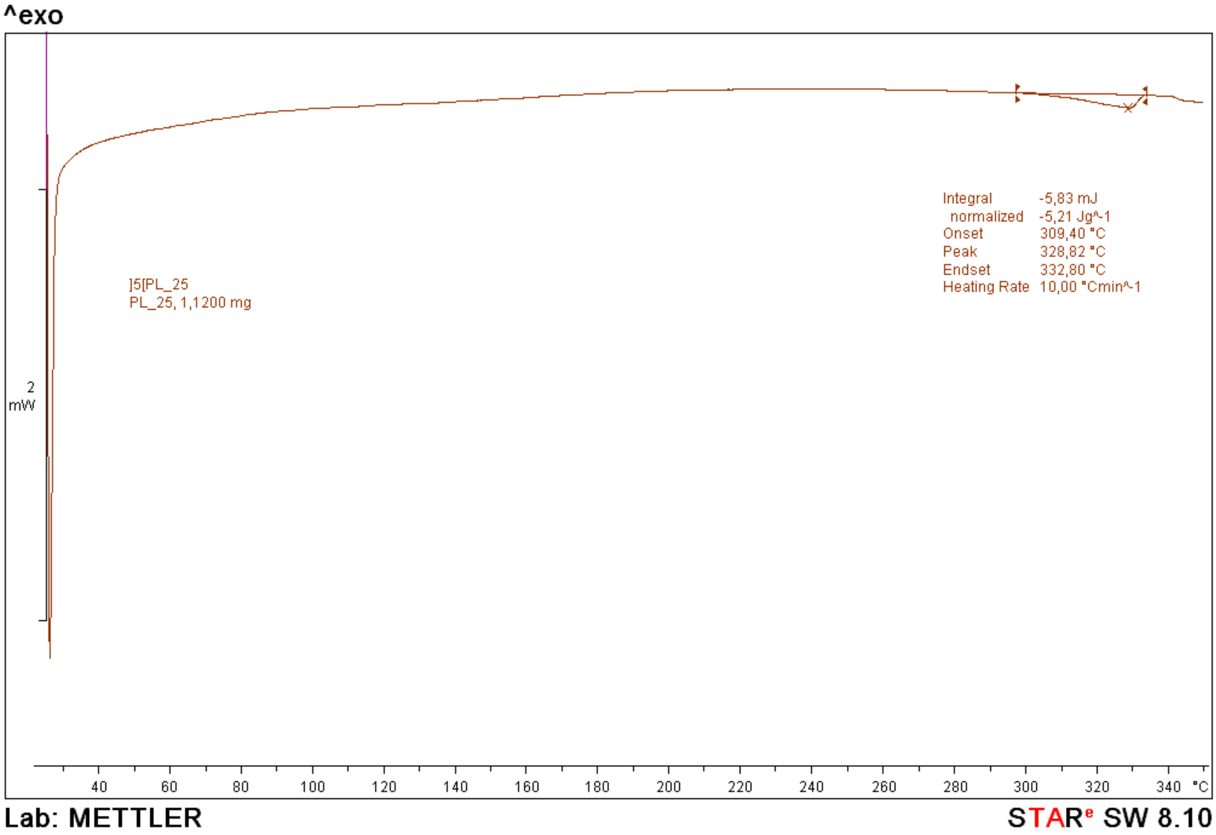

Fig. S2c DSC of PCTB-NDI

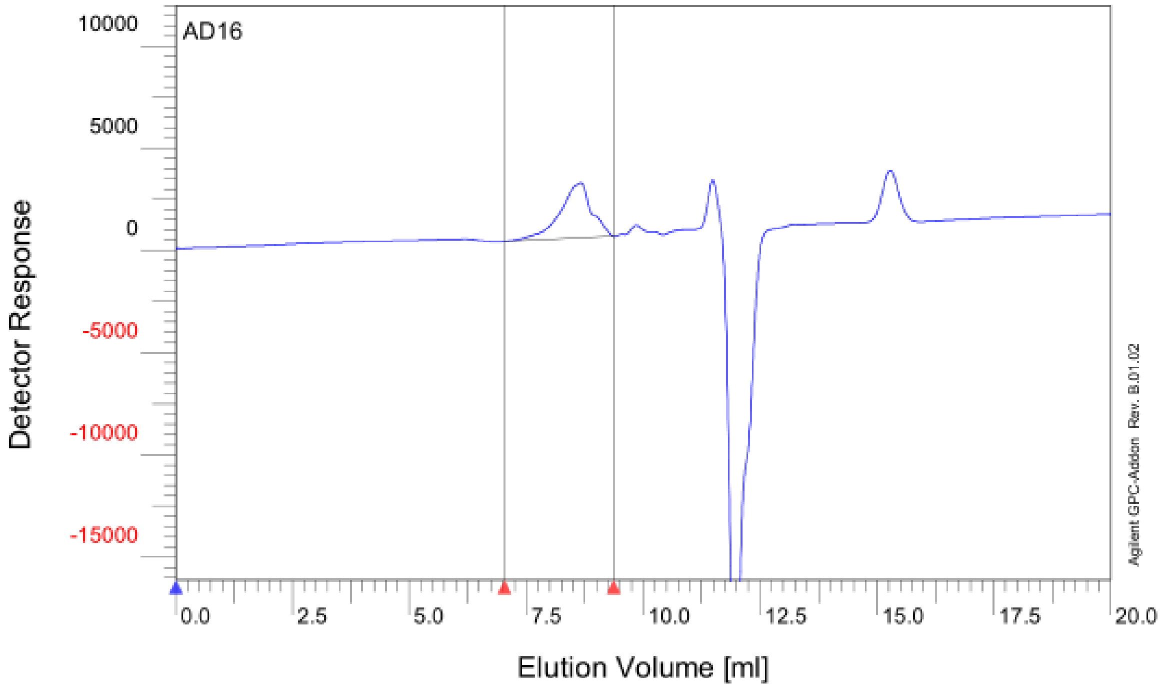

RID1A

**Mn :**	2.9079e3	g/mol
**Mw :**	4.3607e3	g/mol
**Mz :**	7.8963e3	g/mol
**Mv :**	0.000000	g/mol
**D :**	1.4996e0	
**[n]:**	0.000000	ml/g
**Vp :**	8.6688e0	ml
**Mp :**	2.6426e3	g/mol
**A :**	2.0982e3	ml*V
**10%**	1.6222e3	g/mol
**30%**	2.4719e3	g/mol
**50%**	3.2160e3	g/mol
**70%**	4.3774e3	g/mol
**90%**	7.9037e3	g/mol

Fig. S2d SEC of PCTB-PDI

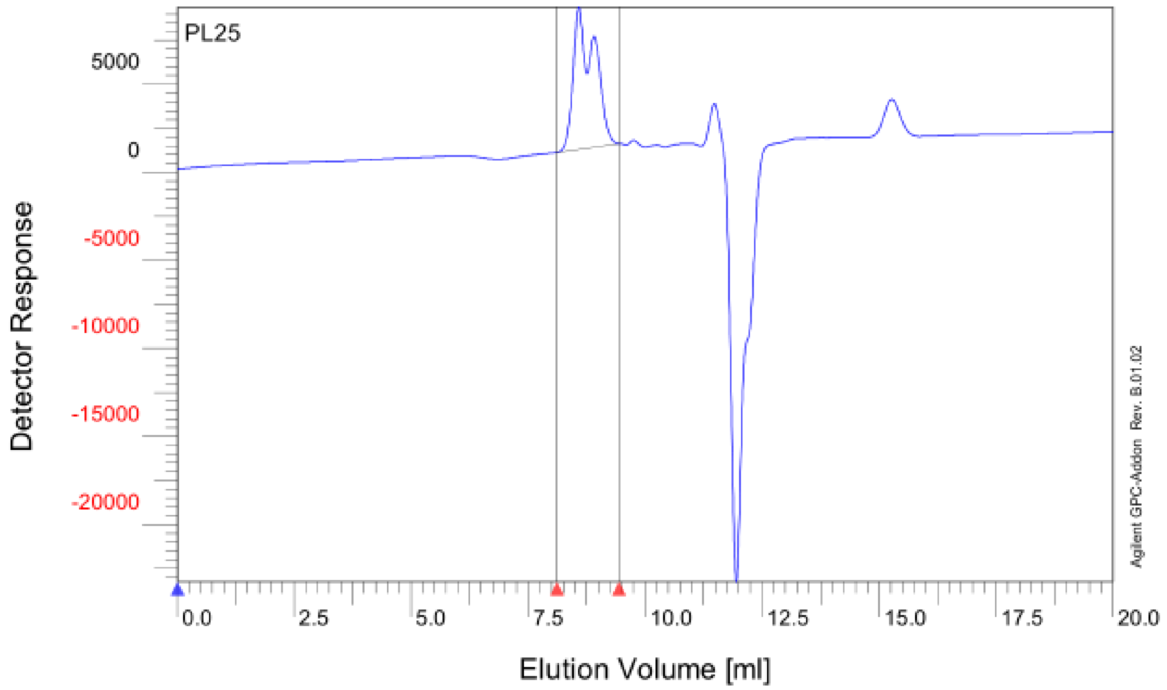

RID1A

**Mn :**	2.1633e3	g/mol
**Mw :**	2.4739e3	g/mol
**Mz :**	2.7861e3	g/mol
**Mv :**	0.000000	g/mol
**D :**	1.1436e0	
**[n]:**	0.000000	ml/g
**Vp :**	8.5823e0	ml
**Mp :**	3.0486e3	g/mol
**A :**	4.1484e3	ml*V
**10%**	1.4191e3	g/mol
**30%**	1.8275e3	g/mol
**50%**	2.4064e3	g/mol
**70%**	2.9892e3	g/mol
**90%**	3.6334e3	g/mol

Fig. S3a SEC of PCTB-NDI

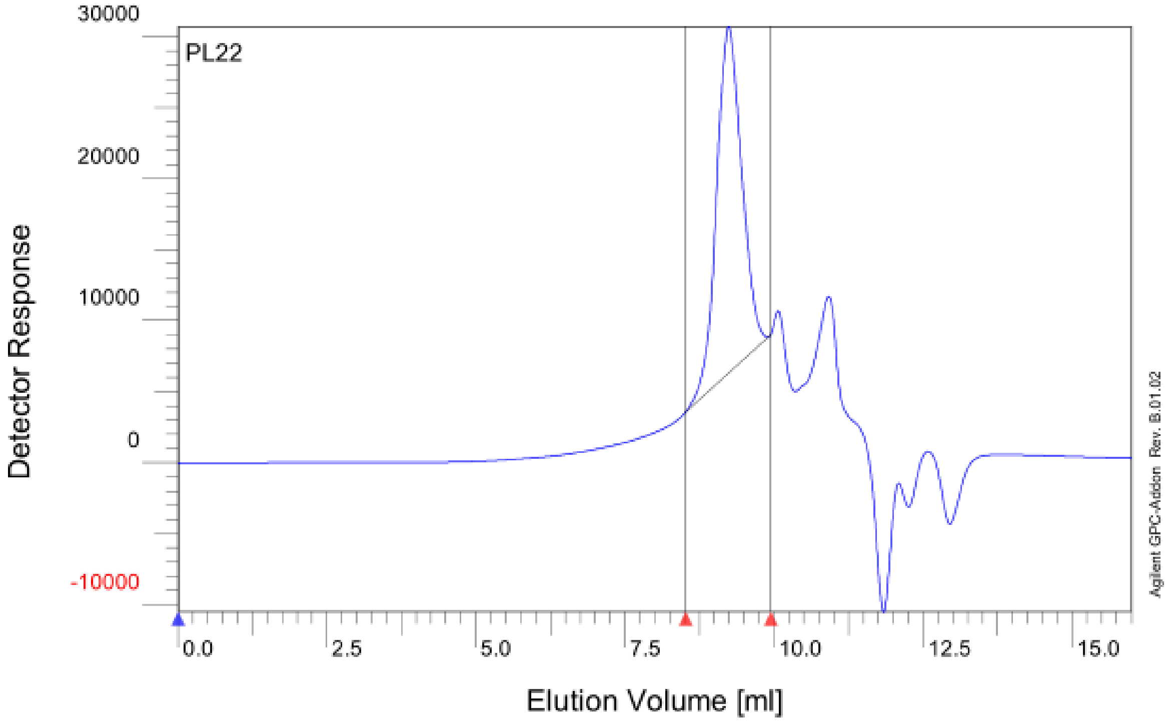

RID1A

**Mn :**	2.2086e3	g/mol
**Mw :**	2.5675e3	g/mol
**Mz :**	3.0168e3	g/mol
**Mv :**	0.000000	g/mol
**D :**	1.1625e0	
**[n]:**	0.000000	ml/g
**Vp :**	9.2333e0	ml
**Mp :**	2.4472e3	g/mol
**A :**	1.1939e4	ml*V
**10%**	1.4447e3	g/mol
**30%**	1.9304e3	g/mol
**50%**	2.3662e3	g/mol
**70%**	2.8965e3	g/mol
**90%**	3.8778e3	g/mol

Fig. S3a SEC of PCTB

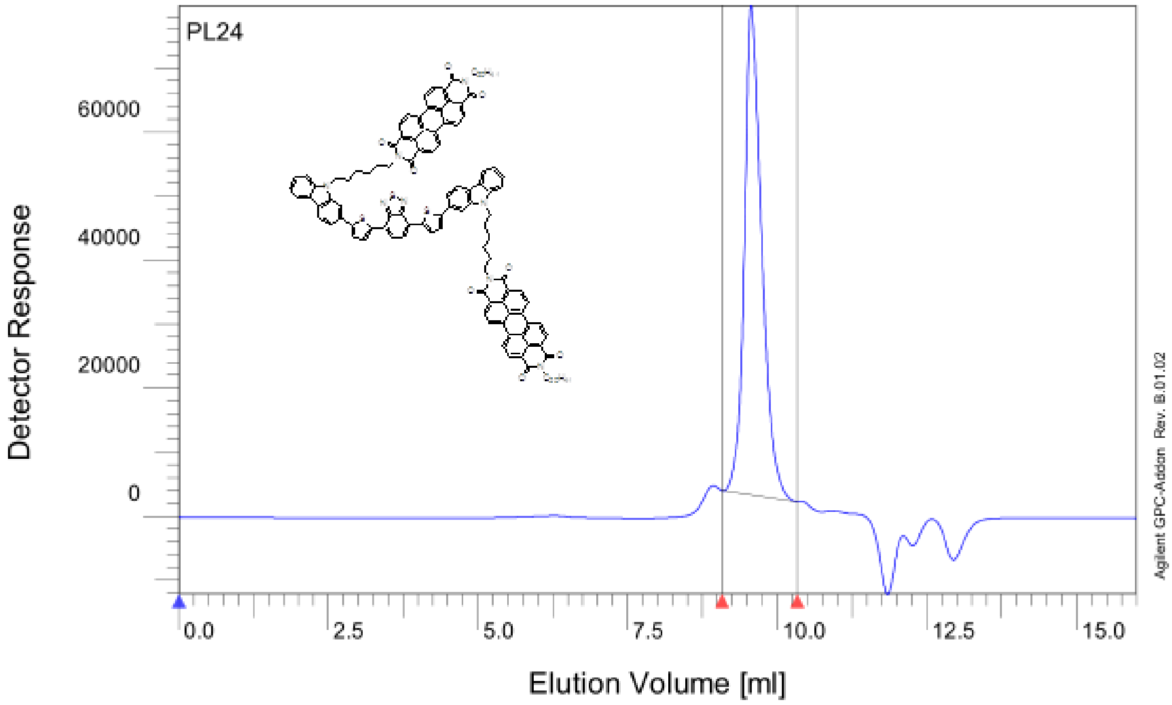

RID1A

**Mn :**	1.1563e3	g/mol
**Mw :**	1.2624e3	g/mol
**Mz :**	1.3545e3	g/mol
**Mv :**	0.000000	g/mol
**D :**	1.0918e0	
**[n]:**	0.000000	ml/g
**Vp :**	9.5616e0	ml
**Mp :**	1.3456e3	g/mol
**A :**	2.5755e4	ml*V
**10%**	8.5004e2	g/mol
**30%**	1.0954e3	g/mol
**50%**	1.2571e3	g/mol
**70%**	1.4131e3	g/mol
**90%**	1.6667e3	g/mol

Fig. S3d SEC of reference oligomer

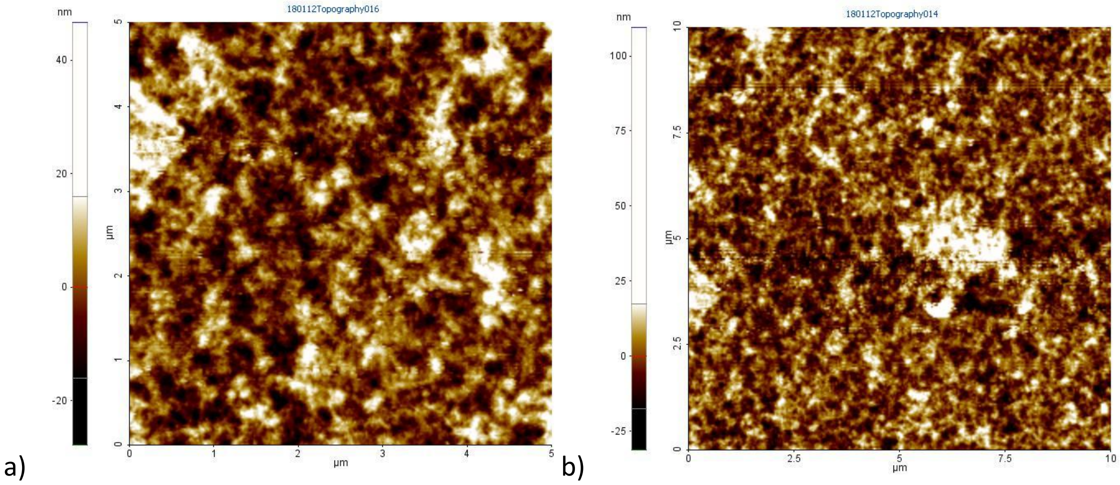

Fig. S4 AFM images of PCTB-PDI
